# Mix of destinations and sedentary behavior among Brazilian adults: a cross-sectional study

**DOI:** 10.1186/s12889-020-10123-7

**Published:** 2021-02-12

**Authors:** Alex Antonio Florindo, Gavin Turrell, Leandro Martin Totaro Garcia, João Paulo dos Anjos Souza Barbosa, Michele Santos Cruz, Marcelo Antunes Failla, Breno Souza de Aguiar, Ligia Vizeu Barrozo, Moises Goldbaum

**Affiliations:** 1 School of Arts, Sciences and Humanities, University of Sao Paulo, Rua Arlindo Bettio, Sao Paulo, SP 1000 Brazil; 2 Graduate Program in Nutrition in Public Health, School of Public Health, University of Sao Paulo, Sao Paulo, Brazil; 3 Physical Activity Epidemiology Group, University of Sao Paulo, Sao Paulo, Brazil; 4 Centre for Research and Action in Public Health, Health Research Institute, University of Canberra, Canberra, Australia; 5 Centre for Public Health, Queen’s University Belfast, Belfast, UK; 6 Department of Epidemiology and Information, Municipal Government of Sao Paulo, Sao Paulo, Brazil; 7 Department of Geography, School of Philosophy, Literature and Human Sciences, University of Sao Paulo, Sao Paulo, Brazil; 8 Department of Preventive Medicine, School of Medicine, University of Sao Paulo, Sao Paulo, Brazil

**Keywords:** Built environment, Mix of destinations, Sedentary behavior, Sitting time, Adults, Brazil

## Abstract

**Background:**

Sedentary behavior is influenced by contextual, social, and individual factors, including the built environment. However, associations between the built environment and sitting time have not been extensively investigated in countries with economies in transition such as Brazil. The objective of this study is to examine the relationship between sitting-time and access to a mix of destinations for adults from Sao Paulo city, Brazil.

**Methods:**

This study uses data from the Health Survey of Sao Paulo. Sedentary behavior was assessed by a questionnaire using two questions: total sitting time in minutes on a usual weekday; and on a usual weekend day. The mix of destinations was measured by summing the number of facilities (comprising bus stops, train/subway stations, parks, squares, public recreation centres, bike paths, primary health care units, supermarkets, food stores, bakeries, and coffee-shops) within 500 m of each participant’s residence. Minutes of sitting time in a typical weekday and weekend day were the outcomes and the mix of destinations score in 500 m buffers was the exposure variable. Associations between the mix of destinations and sitting time were examined using multilevel linear regression: these models accounted for clustering within census tracts and households and adjusted for environmental, sociodemographic, and health-related factors.

**Results:**

After adjustment for covariates, the mix of destinations was inversely associated with minutes of sitting time on a weekday (β=− 8.8, *p*=0.001) and weekend day (β=− 6.1, *p*=0.022). People who lived in areas with a greater mix of destinations had shorter average sitting times.

**Conclusion:**

Greater mix of destinations within 500 m of peoples’ residences was inversely associated with sitting time on a typical weekday and weekend day. In Latin American cities like Sao Paulo built environments more favorable for walking may contribute to reducing sedentary behavior and prevent associated chronic disease.

## Background

Sedentary behavior - i.e. extended periods of sitting timing - is a significant public health problem: it accounts for 3.8% of all-cause mortality [1], and increases the risk of cardiovascular disease and diabetes, and premature mortality [2–4]. In addition, sedentary behavior is associated with loss of functional capacity and daily life activities and poorer quality of life in elderly people [5], with weight gain from childhood to adulthood [6], and with an unhealthy diet and food consumption [7].

Sedentary behavior is associated with different correlates measured at intrapersonal, social, physical environmental, and policy levels [8]. Early studies examining relationships between the built environment and sedentary behavior show that low walkability was associated with more television viewing in Australian females [9], and in North American adults [10]. A study conducted in 11 cities with 5.712 adults which measured sedentary behavior using accelerometers found that greater street connectivity and a more diverse land use mix were associated with fewer minutes per day of sedentary time; and higher residential density and higher pedestrian infrastructure were associated with more minutes per day of sedentary time [11]. A systematic review showed that access and proximity to general services and facilities and recreation facilities were inversely associated with total sitting time [12]. Another cross-sectional study showed that the walkability index calculated on the basis of land use mix, street connectivity, and residential density, was positively associated with sedentary behavior in Belgian adults [13]. However, other systematic reviews have found limited evidence for an association between sitting time and access to destinations, land use mix, and street connectivity [8]. Therefore, studies examining the association between the built environment and sedentary behavior have produced inconsistent and inconclusive evidence, and relationships might differ depending on the domain of sedentary behavior being investigated [14].

In addition, we have few studies in low and middle-income countries that describe the relationship between the built environment and sedentary behavior. For example, the study of Owen et al. [11] involved adults from Curitiba, Brazil, and Bogota, Colombia. The low and middle-income countries are economies in transition and their built environments are different from those found in high-income countries. Therefore, the objective of this study is to examine the relationship between the mix of destinations and total sitting time in adults from Sao Paulo city, a densely populated Latin American megalopolis in a middle-income country (Brazil).

## Methods

### Health survey of Sao Paulo

This study used data from the Health Survey of Sao Paulo or *Inquérito de Saúde de São Paulo – ISA* in portuguese. Data collection was completed in 2015, with 4043 participants who lived in five health administrative areas in Sao Paulo city. The sampling process has been described in more detail elsewhere [15]. Briefly, the survey used a multi-stage sampling design: from the five health administration areas in Sao Paulo, 150 census tracts were randomly selected, and then households were sampled randomly from each tract. The data were collected using face-to-face interviews in households. The interviews were conducted between September 2014 and December 2015, and 73.4% of eligible residents who were contacted agreed to participate [15].

Georeferencing resulted in 3145 participants aged 18 years or more having their residential address geocoded [16]. More details can be obtained from other publications [16–18]. The ISA forms the baseline dataset for a recently funded longitudinal study of the “ISA-Physical Activity and Environment Study”, which is being conducted among adults in Sao Paulo city, Brazil. A key focus of this prospective research will be to verify the robustness of the cross-sectional studies that have been conducted using the Sao Paulo Health Survey baseline.

### Sedentary behavior

Sedentary behavior data were collected using the International Physical Activity Questionnaire (IPAQ) [19] and measured on the basis of two questions: 1) Total sitting time on a usual weekday; 2) Total sitting time on a usual weekend day. For analysis, two outcomes were used: 1) Continuous measures of minutes of sitting time on a typical weekday; and 2) Continuous measures of minutes of sitting time on a typical weekend day.

### Mix of destinations

Walkable destinations within each participant’s residential catchment were captured using georeferencing procedures [16–18] applied to publicly available datasets, and included eleven destinations: 1. Bus stops; 2. Train/subway stations; 3. Parks; 4. Squares; 5. Public recreation centres; 6. Bike paths; 7. Primary health care units; .8 Supermarkets; 9. Food stores; 10. Bakeries; and 11. Coffee shops. The dataset for items 1 to 8 pertain to places in 2016 and was obtained mainly from the open site GEOSAMPA <http://geosampa.prefeitura.sp.gov.br/PaginasPublicas/_SBC.aspx>, and items 9 to 11 were sourced from the Health Surveillance Registration database from Sao Paulo city associated with the National Economic Activity Classification in November 2016.

We calculated a measure of destination diversity in three phases: 1) by firstly summing the number of each destination within a 500 m radial buffer of each participant’s home address; 2) by secondly, we categorized the participants into two groups based on the sum for each destination. The participants that were at or below the sample median had scored 0, and participants were above the median had scored 1; 3) and thirdly, by the sum of all destination obtained in the second phase we created the mix destination score that ranged from 0 to 8 (mean=3.07, SD=1.70, median=3 interquartile range: 4 ;2). This process has been described in more detail elsewhere in a study also used the Sao Paulo Health Survey which showed that the mix of destination score within a 500 m buffer was significantly associated with walking for transport [18].

### Covariates

We used age (18–29 years, 30–39 years, 40–49 years, 50–59 years, 60 years or more), education (incomplete elementary school, incomplete high school, complete high school, incomplete undergraduate or above), marital status (singles, married/with partners, separated/widowers), obesity (in two categories: BMI < 30 kg/m^2^ or above), physical activity (< 150 min per week or above evaluated by IPAQ long form) [19], self-report of diseases diagnosed by physicians (none or at least one of the following: hypertension; diabetes; myocardial infarction; cardiac arrhythmia; other heart disease; cancer; arthritis, rheumatism or arthrosis; osteoporosis; asthma or asthmatic bronchitis; emphysema, chronic bronchitis or chronical obstructive pulmonary diseases; rhinitis; chronic sinusitis; other lung disease; tendonitis, repetitive strain injury or work-related musculoskeletal disorders; cerebral vascular accident or stroke; spine disease or spine problem), smoking status (yes or no), car or motorcycle ownership (yes or no); time living in the same residence (< 1 year, ≥1 year or < 5 years, > 5 years), and region where people lived in Sao Paulo city (North, South, Midwest, Southeast, and East). These covariates were selected based on the findings of systematic reviews about sedentary behavior correlates in adults [8, 12, 14] and in another original study that examined the relationship between walking for transportation and built environment variables [18].

### Statistical analysis

For this study, we excluded from the analyses people who reported zero minutes of sitting time, those who did not answer the sitting time question in the survey, and those with missing data on the covariates. These exclusions resulted in a final analytic sample of *n*=3052 participants for sitting time on a typical weekday, and *n*=2993 participants for sitting time on a typical weekend day. The analyses are conducted in two stages. First, we present mean sitting times for each of the sociodemographic, health, and environmental covariates, and for participants who were grouped into the two destination-mix categories based on the median split. Second, we examine the multivariable association between the destination mix index and sitting time using multilevel linear regression without and with adjustment for the covariates. The multilevel analysis accounted for clustering within census-tracts and households. All analyses were conducted using Stata version SE 12.1. (StataCorp LP, College Station, USA). We used the xtmixed command for linear models and the results are presented as beta coefficients (β) with 95% confidence intervals.

### Ethics approval

The Ethics Committee of the School of Arts, Sciences, and Humanities at the University of Sao Paulo approved the study (process number 55846116.6.0000.5390).

## Results

Mean sitting time during weekdays was higher than weekends (Table 1). Mean sitting time was higher for men, persons aged 18–29, the highly educated, those who engaged in insufficient physical activity, classified as obese, who reported having at least one disease, single, who owned a private motor vehicle, living in the Midwest region of the city, and who had lived at their current residence for less than one year.

**Table 1:** Descriptive statistics for sitting time on a typical weekday and weekend day by social, demographic, health, and environmental variables, Sao Paulo city, Brazil, 2015

	Minutes of sitting time on a typical day	
	Weekdays	Weekend days
	n=3052	n=2993
	mean (SD)	mean (SD)
Overall	279.8 (199.2)	260.5 (183.5)
Gender		
Men	295.3 (203.7)	274.7 (191.5)
Women	268.3 (195.1)	249.8 (176.5)
Age		
18–29	347.0 (217.7)	281.7 (200.5)
30–39	277.1 (203.9)	255.8 (179.6)
40–49	255.7 (187.7)	242.9 (172.9)
50–59	244.0 (177.8)	241.2 (163.6)
60 or more	260.5 (185.6)	265.1 (184.7)
Education		
Incomplete elementary school	240.1 (195.8)	256.2 (197.9)
Incomplete high school	249.9 (187.1)	248.5 (180.1)
Complete high school	282.8 (194.1)	255.1 (169.3)
Undergraduate incomplete or more	353.1 (202.6)	285.2 (185.4)
Physical activity		
≥ 150 min per week	261.8 (187.0)	243.7 (170.0)
< 150 min per week	354.8 (229.2)	325.7 (214.9)
Body Mass Index (kg/m^2^)		
≥30 kg/m^2^	294.4(195.2)	279.9(183.9)
< 30 kg/m^2^	275.9(199.4)	255.6(181.8)
Presence of diseases		
Yes	282.5 (198.3)	268.1 (188.5)
No	272.8 (199.4)	244.4 (173.3)
Smoking status		
Yes	284.5 (203.3)	270.9 (196.1)
No	279.0 (198.5)	258.5 (181.0)
Marital Status		
Married, or with partners	262.2 (190.1)	251.4 (176.3)
Singles	322.6 (214.6)	275.3 (193.9)
Separated, or widowers	270.0 (189.9)	264.5 (183.1)
Car or motorcycle ownership		
Yes	288.9 (197.5)	260.7 (175.8)
No	268.6 (200.7)	260.3 (192.5)
Region of residence		
North	284.0 (212.4)	262.2 (197.2)
South	272.0 (180.8)	247.2 (168.7)
Midwest	311.9 (198.0)	285.3 (184.5)
Southeast	258.0 (204.5)	247.5 (183.4)
East	268.3 (191.4)	254.9 (177.6)
Length of residence		
< 1 year	300.3 (212.7)	288.9 (206.3)
> 1 year and < 5 years	285.8 (203.8)	247.7 (172.4)
> 5 years	275.6 (195.6)	259.5 (182.4)

SD (standard deviations)

**Fig. 1:**
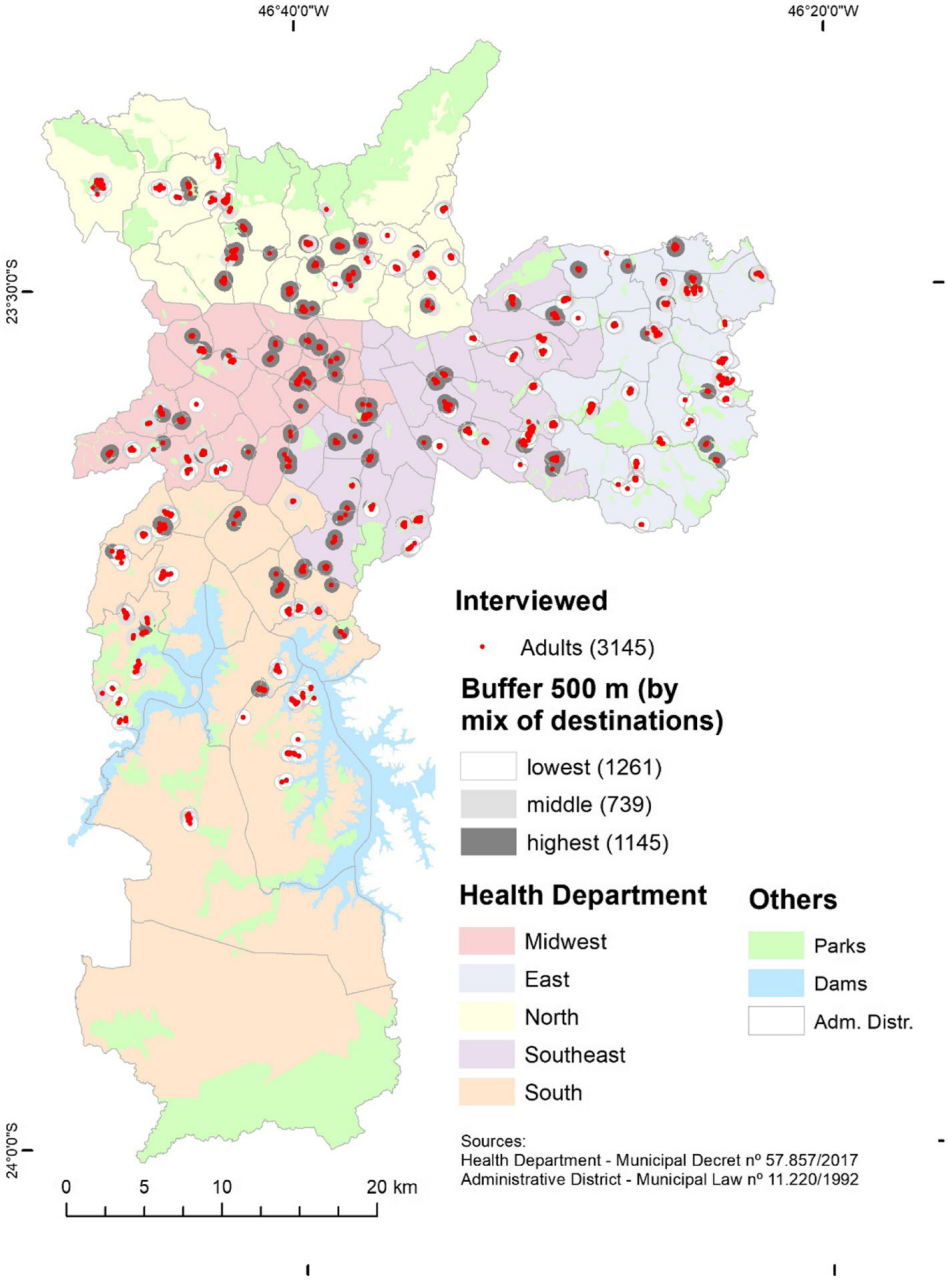
Destinations mix according to health administration area where people reside in Sao Paulo city, Brazil, 2016. Shapefiles of Health Departments are provided by the Municipal Health Secretariat. The shapefile of the Administrative Districts was furnished by the Municipal Secretariat for Urban Development. Both are not under license and publicly available at http://geosampa.prefeitura.sp.gov.br/PaginasPublicas/_SBC.aspx#. The map was created with the software ArcGIS Desktop 10.7, version 10.7.0.10450, Copyright (C)1999–2018 Esri Inc.

Buffers with the highest concentration of destinations were found in the central areas of the city (Fig. 1) and sitting time was higher in places with the lowest mix of destinations (Table 2).

**Table 2:** Descriptive statistics and bivariate analysis for sitting time according to mix of destination scores, Sao Paulo city, Brazil

	Minutes of sitting time		
	Typical weekdays		Typical weekend days
Destination mix scores	Mean (SD)		Mean (SD)
At or below the median*	290.1 (206.0)		271.6 (191.0)
Above the median*	273.0 (194.3)		253.1 (178.0)
p-value**	0.039		0.014

* Median score for mix of destination within each 500 m buffer; **p values based on a Kruskal-Wallis test; SD (standard deviations)

There was a statistically significant association between the mix of destinations score and minutes of sitting on a typical weekday and weekend day after adjustment for the covariates (Table 3). For each point increase in destination score mix, we had a mean decrease in 8.8 min of sitting time on a typical weekday and a mean decrease in 6.1 min of sitting time on a typical weekend day.

**Table 3:** Multilevel linear regression results examining the association between mix of destinations* and minutes sitting on a typical weekday and weekend day among adults from Sao Paulo City, Brazil

	Unadjusted			Adjusted**		
	β	95%CI	p value	β	95%CI	p value
**Minutes on a typical weekday**						
Destination mix score*	−4.63	−9.94, 0.66	0.086	−8.83	−14.27, −3.38	0.001
**Minutes on a typical weekend day**						
Destination mix score*	−4.89	−9.91, 0.13	0.056	−6.10	−11.32, −0.89	0.022

*Destination mix was measured using an index, which ranged from 0 to 8 (mean 3.07, SD, median 3.0)

**Adjusted for gender, age, education, marital status, obesity, physical activity, smoking, disease presence, car or motorcycle ownership, region of residence in Sao Paulo, and length of residence at the surveyed address

## Discussion

The main result of this study showed that after adjustment for sociodemographic, environmental, and health-related factors, people with a greater mix of destinations within 500 m of their residence reported engaging in fewer minutes of sitting time on a typical weekday and weekend day.

The results of this study are consistent with the findings of previous research examining the relationship between the mix of destinations and sedentary behavior. A systematic review showed that access to destinations was inversely associated with sitting time, particularly access to leisure and transportation destinations [14]. A cross-sectional study conducted with Japanese adults living in Tokyo found indicative (*p*-value = 0.051) results that access to 30 or more different types of destinations might be inversely associated with sitting time when using transportation to access leisure activities [20]. However, a longitudinal study with adults from Nerima and Kanuma cities in Japan did not find an association between screen time and access to different types of destinations [21]. In addition, studies conducted with adults from high-income countries which have used accelerometers to measure sedentary behaviour have either found no association with built environment variables [22, 23] or that people living in areas with higher walkability engage in more minutes of sitting time [13], a result that was contrary to expectations.

The mix of destinations measure used in this study included access to green space, physical activity, and transport nodes, primary health care units, supermarkets, and other shops, within 500 m of each participant’s residence. In a previous study, also using the Health Survey of Sao Paulo sample, it was found that a greater mix of destinations close to home was associated with an increased likelihood of walking [18]; and a systematic review reported that physical activity was inversely associated with sitting time in adults [24].

The results of this study are important as very few studies of the built environment and sedentary behavior have been conducted in densely populated Latin American cities. In Sao Paulo city, the local government has introduced policies such as a New Master Plan to address environmental inequities, increase physical activity, and reduce sedentary behavior [25].

This study had several limitations that should be considered when interpreting the results. Firstly, sedentary behavior was measured by self-report. Sitting time is a complex behavior for people to recall accurately because it is necessary to consider all domains of life (work, household, leisure, and transportation). In this case, underestimation due to measurement error is likely to be present in our findings [8, 26]. Secondly, it is important to repeat our study using longitudinal data with residentially stable participants as a way of adjusting for bias that results from neighborhood self-selection (e.g. less sedentary people moving to neighborhoods with more walkable destinations). For example, a recent systematic review showed that obesity was inversely associated with walkability in cross-sectional studies but not in longitudinal studies [27]. Thirdly, the findings of this study may have differed had we examined the determinants of sitting time in specific domains of sedentary behavior such as leisure, work, transport, and within the household [8, 12, 26].

## Conclusion

A more diverse mix of walkable destinations within 500 m of adults’ homes in Sao Paulo City was associated with fewer minutes of sitting time on a typical weekday and weekend day. These results suggest that city planners and urban designers have an important public health role to play in helping to reduce sedentary behaviour, promote physical activity, and prevent associated chronic disease in Latin American countries with economies in transition such as Brazil.

## Data Availability

The datasets used during the current study are available from Sao Paulo Health Survey. For permissions to access the data to request for professor Regina Mara Fisberg by email or phone call, School of Public Health at University of Sao Paulo, Brazil, email: rfisberg@usp.br, phone: + 55 11 3061–7701.

## Abbreviations

IPAQ: International Physical Activity Questionnaire
ISA: Health Survey of Sao Paulo
CI: Confidence interval
OR: Odds Ratio
